# Restoration of LRIG1 suppresses bladder cancer cell growth by directly targeting EGFR activity

**DOI:** 10.1186/1756-9966-32-101

**Published:** 2013-12-08

**Authors:** Lei Chang, Runlin Shi, Tao Yang, Fan Li, Guohao Li, Yonglian Guo, Bin Lang, Weimin Yang, Qianyuan Zhuang, Hua Xu

**Affiliations:** 1 Department of Urology, Tongji Hospital, Tongji Medical College, Huazhong University of Science and Technology, Wuhan 430030, China; 2 Institute of Urology, Tongji Hospital, Tongji Medical College, Huazhong University of Science and Technology, Wuhan 430030, China; 3 Department of Urology, Central Hospital of Wuhan, Wuhan 430030, China; 4 School of Health Sciences, Macao Polytechnic Institute, Macao, China

**Keywords:** LRIG1, EGFR, Apoptosis, Invasion, Bladder cancer

## Abstract

**Background:**

Recently, leucine-rich repeats and immunoglobulin-like domains 1 (LRIG1), a negative regulator of EGFR, was discovered is a novel agent for suppressing bladder cancer. The aim of this study was to investigate the impact of LRIG1 on the biological features of aggressive bladder cancer cells and the possible mechanisms of enhanced apoptosis induced by upregulation of LRIG1.

**Methods:**

In this study, we examined the mRNA and protein expression of LRIG1 and EGFR in bladder cancers and normal bladder. Meanwhile, we overexpressed LRIG1 with adenovirus vector in T24/5637 bladder cancer cell lines, and we used real time-PCR, western blot, and co-immunoprecipitation analysis in order to examine the effects of LRIG1 gene on EGFR. Furthermore, we evaluate the impact of LRIG1 gene on the function of human bladder cancer cells and EGFR signaling.

**Results:**

The expression of LRIG1 was decreased, while the expression of EGFR was increased in the majority of bladder cancer, and the ratio of EGFR/LRIG1 was increased in tumors versus normal tissue. We found that upregulation of LRIG1 induced cell apoptosis and cell growth inhibition, and further reversed invasion in bladder cancer cell lines in vitro by inhibiting phosphorylation of downstream MAPK and AKT signaling pathway.

**Conclusion:**

Taken together, our findings provide us with an insight into LRIG1 function, and we conclude that LRIG1 evolved in bladder cancer as a rare feedback negative attenuator of EGFR, thus could offer a novel therapeutic target to treat patients with bladder cancer.

## Introduction

Bladder cancer is the fourth most common cancer in men after prostate, lung, and colorectal cancers, accounting for 7% of all cancer case [1]. The majority of bladder tumors (75%) are non muscle-invasive at diagnosis and after local surgical therapy, have a high risk of recurrence and a propensity to progress in grade or stage [2]. At present, its major treatment is surgical removal but, with surgical approach, recurrence tends to take place. Muscle invasive tumors (25%) have a poorer prognosis [3] since 50% of patients will relapse with metastatic disease within 2 years of treatment. Patients presenting with muscle invasive cancer or progressing to this stage have a poor survival rate, despite receiving conventional therapies [4]. With the development of the molecular biology, genes involved in tumorigenesis have been targeted for the treatment of tumor.

Epidermal growth factor receptor(EGFR) is a transmembrane protein tyrosine kinase and over-expressed or activated in a variety of malignant lesions, including bladder cancer [5]. Over-expressed or activated EGFR signaling is the initial step of a cascade of events leading to tumor cell proliferation, invasion, migration and evasion of apoptosis [6,7]. Inhibition of EGFR by different approaches causes increased apoptosis and sensitizes tumor cells to radiation therapy and chemical therapy [8,9]. Owing to the important role of the EGFR activation in bladder cancer growth and progression, therefore, it is a potential target for molecular therapy for invasive bladder cancer.

The human LRIG gene family comprises three paralogous genes, namely LRIG1 (formerly LIG1) [10], LRIG2 [11] and LRIG3 [12]. Leucine-rich repeats and immunoglobulin-like domains 1(LRIG1) is a transmenbrane leucine-rich repeat and immunoglobulin(Ig)-like domain-containing protein, whose transcript is located at chromosome 3p14.3, a region frequently deleted in various types of human cancers [10]. It is capable of interacting with EGFR and enhancing both its basal and ligand-stimulated ubiquitination and degradation [13,14]. These reports suggest that LRIG1 is a candidate suppressor of EGFR activity. Previous studies showed that upregulation of LRIG1 expression in the superficial bladder cancer BIU-87 cell lines resulted in inhibition of cell proliferation and attenuation of cell invasive abilities, and played a tumor-suppressive role in vivo in bladder cancer [15,16]. But the impact of LRIG1 on the biological behaviors of aggressive bladder cancer cells in vitro and the possible mechanisms of enhanced apoptosis induced by upregulation of LRIG1 is not very clear.

In this study, we observed that LRIG1 expression appeared significantly downregulated, but EGFR markly elevated in the majority of bladder cancer compared to human normal bladder tissue. Upregulation of LRIG1, followed by a decrease of EGFR on protein expression, induces cell apoptosis and cell growth inhibition, further reversing invasion in aggressive bladder cell lines. Finally, we demonstrated the capacity of upregulation of LRIG1 to inhibit downstream EGFR signaling in bladder cancer cells as manifested by markedly decreased expression of p-MAPK and p-AKT. Taken together, we conclude that restoration of LRIG1 to bladder cancer could offer a novel therapeutic strategy for suppression of receptor-positive bladder cancer.

## Materials and methods

### Tissue samples

All of the tissue specimens were obtained between November 2011 and September 2012 from 50 patients who underwent surgery for therapeutic treatment at Tongji Hospital. Immediately after the surgery, samples were snap-frozen in liquid nitrogen and stored at -80°C. There were 45 bladder cancer and 5 normal bladder tissues in all of the specimens. As controls, biopsies of normal bladder samples were obtained from 5 patients who underwent transvesical prostatectomy. No treatment was given to the patients before surgery. The samples were sectioned for hematoxylin and eosin (H&E) staining for histological confirmation by the Department of Pathology of Tongji hospital. Tumor staging was determined according to the sixth edition of the tumor node metastasis (TNM) classification of the International Union Against Cancer. This study was approved by the ethnics committee of Huazhong University of Science and Technology. All patients provided informed consent.

### Reagents and cell culture

The plasmid p3XFLAG-CMV9-LRIG1 and rabbit antihuman LRIG1 polyclonal antibodies were generous gifts from Hakan Hedman (Umea University, Sweden). Two human aggressive bladder cancer cell lines(T24 and 5637) were used in this study. All of this cell lines were obtained from the American Type Cell Collection(ATCC), and grown in complete growth medium supplemented with 10% fetal bovine serum(FBS) and maintained in a humidified 5% CO_2_ atmosphere 37°C.

### Cell transfection

The plasmid p3XFLAG-CMV9-LRIG1 was transfected into the two bladder cancer cells by using Lipofectamine2000 reagent (Invitrogen, Groningen, the Netherlands) according to the manufacturer’s instructions. For control experiments, the vector p3XFLAG-CMV9-EGFP was also transfected into the two bladder cancer cells. All transfected cells were exposed to G418 (800 μg/mL, Sigma Chemical Co., St. Louis, USA) for 3 weeks of selection. Resistant clones representing stably transfected cells were ring-cloned and expanded for further experiment.

siRNAs against EGFR were transfected into T24 and 5637 cells according to the transfection protocol of Lipofectamine2000 (Invitrogen). A nonspecific control siRNA strand was used as a negative control. Seventy-two hours after transfection, knockdown was assessed by western blot from a parallel transfection. After downregulation of EGFR, we detected the effect of LRIG1 cDNA on cell proliferation and EGFR signaling pathway by CCK-8 assays and western blot respectively.

### Quantitative real-time RT-PCR

Total RNA was extracted from 45 cases of bladder cancer and 5 cases of respective non-neoplastic tissue samples and 2 bladder cancer cell lines with Trizol reagent. The expression of LIG1 and EGFR mRNA was done using quantitative real-time RT-PCR. RNA samples were run in triplicate using 20 ng of RNA perreaction. The resulting cDNA samples were amplified by real-time PCR using gene-specific primer sets in conjunction with the SYBR Premix Ex Taq (TaKaRa) in a Mx3000p instrument. The qPCR was performed with the following conditions: activation at 95°C for 5 min followed by 40 cycles of denaturation at 94°C for 15 s, amplification at 60°C for 30 s, elongation at 72°C for 30 s. In the last, a cycle of solubility curve was added to examine the amplification quality. Expression of mRNA for GAPDH was used as an internal standard. Reverse transcription products were amplified by PCR using specific primers for human LRIG1 (forward 5′-GGTGAGCCTGGCCTTATGTGAATA-3′; reverse 5′-GGTGAGCCTGGCCT TATGTGAATA-3′) and human EGFR (forward 5′-TCCCTCAGCCACCCATAT GTAC-3′; reverse 5′-TCCCTCAGCCACCCATATGTAC-3′).

### Immunohistochemistry(IHC)

Formalin-fixed and paraffin-embedded tissue sections (5 mm) were dewaxed with xylene and rehydrated through an ethanol gradient into water. Following blocking of endogenous peroxidase activity with 0.3% hydrogen peroxide for 10 min, the sections were washed with phosphate buffered saline(PBS) and incubated over-night with rabbit LRIG1 antibody or EGFR antibody at the dilution of 1:100 in a humidified chamber at 4°C. After washing with PBS, sections were incubated with biotinylated secondary antibody for 30 min at 37°C and then with horseradish peroxidase labeled streptavidin for 30 min at 37°C. Diaminobenzidine(DAB) was used as chromogen and the sections were subsequently counterstained with hematoxylin, then dehydrated, cleared and mounted.

### Western blotting analysis

The transfected bladder cancer cells were collected and washed with 0.01 mol/L PBS for three times. Then the cells were added into 200ul pre-cold RIPA-PICT cell disruption liquor and centrifuged. All subsequent manipulations were performed on ice. After centrifugation, the supernatant was collected. The protein concentration of each sample was measured with micro-BCA protein assay reagent. The mixture was heated to 100°C for 5 min to denature the proteins. The protein from each sample was subjected to electrophoresis on 10% sodium dodecyl sulfate–polyacrylamide gel. Then protein was transferred to nitrocellulose membrane, which were blocked with PBS containing 5% non-fat milk for 2 h and then incubated with anti-LRIG1 (1:5,000), anti-EGFR(1:2,000), anti-p-EGFR(1:2,000), anti-MAPK(1:2,000), anti-p-MAPK(1:2,000), anti-AKT(1:2,000), anti-p-AKT(1:2,000), anti-caspase-8(1:1,000), anti-MMP-2(1:2,000), anti-MMP-9(1:2,000) and β-actin(1:2,000) at 4°C overnight. Then secondary antibody labeled with alkaline phosphatase were added at room temperature. One hour later, the samples were washed for three times with TBST, and then visualized using DAB detection system.

### Immunoprecipitation

The total protein was prepared using M-PERTM mammalian protein extraction reagent (Pierce). For each sample, 10 μL of anti-LRIG1 antibody or control IgG was added to 1 mg of protein in 200 μL of lysis buffer and placed on a rocker overnight at 4°C. Twelve microliters of protein G beads was added to each sample, which was placed on a rocker at 4°C for 1 h. The beads were washed three times with 1 ml of lysis buffer and then boiled in 50 μL of SDS sample buffer; 20 μL was then loaded per lane and subjected to Western blotting.

### Apoptosis analysis

Annexin V-PE/7-aad double staining assay was used to detect cell apoptosis. After transfected and incubated for 3 days, cells were collected, centrifuged and washed with phosphate—buffered saline(PBS) for two times. Binding buffer was then added to each tube and cells were re-suspended. The cells were incubated with 5 μL of annexin V-PE and 5 μL of 7-aad for 15 min at room temperature in the dark. Then, the apoptotic analyses were done by flow cytometry within one hour.

### Survival assay by CCK-8

The growth of T24 and 5637 cells after LRIG1 gene transfection were evaluated by Cell Counting Kit-8 assays. Untreated cells, cells treated with liposome alone and cells treated with the vector control were used for comparison. Cell suspensions (at 1 × 10^3^/mL) were transferred to 96-well plates in triplicate and incubate for 24, 48 and 72 hours. Subsequently, CCK-8(10 μL) was added to each well, cells were incubated for an additional 4 h. Then, The values of each well was measured by microplate reader at 450 nm.

### Clonal forming assay

T24 and 5637 cells were infected with LRIG1 cDNA and cultured for 24 h, then plated in 6-well plates at 200 cells/well. Plates were subsequently incubated for 14 days in a humidified incubator at 37°C, and the colonies were stained with 0.5 ml of 0.0005% crystal violet solution for 1 h and counted by using a microscope. Five random fields were counted from each sample and average values presented ± the SD.

### Matrigel invasion assays

The in vitro invasive ability of bladder cancer cells was measured in transwells chambers assay. 100ul matrigel was put into upper chambers of the transwell insets. Incubated the inserts at 37°C for 4 h for gelling and then pretreated with serum-free medium at 37°C for 1 h before seeding cells at a density of 2 × 10^4^ /ml with 1% FCS. The lower chambers of the transwells were filled with 600 ul medium containing 10% FCS. Then the transwell were incubated at 37°C with 5% CO_2_ for 24 h to allow cells to migrate. After that, removed the cells on the upper side by wiping with cotton swab. Cells that had invaded through matrigel were fixed in paraformaldehyde and crystal violet stained according to the manufacture’s instruction. Cells that had invaded the matrigel and reached the lower surface of the filter were counted under a light microscope at a magnification of 200×. We chose five fields of vision and counted the numbers of the invaded cells and the results from three separate chambers were then averaged. The experiment was performed in triplicate.

### Statistical analysis

The cell culture data from at least three independent experiments were expressed as means ± SD and examined by one-way analysis of variance followed by the Student–Newman–Keuls test. A Pearson’s correlation test was performed to examine the relationship of LRIG1 and EGFR expression in bladder cancer and non-neoplastic tissues. All P-values were two-sided, and values less than 0.05 were considered significant. SPSS v16.0 software was used for all statistical procedures.

## Results

### Expression of LRIG1 and EGFR mRNA and protein in bladder cancer and normal tissue

**Figure 1:**
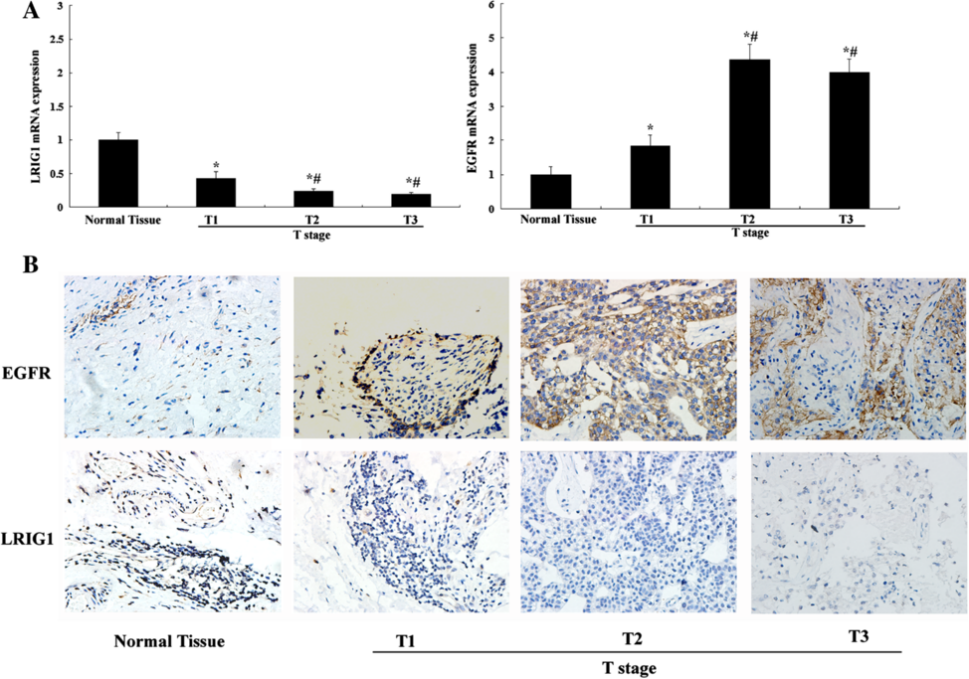
**Expression of LRIG1 and EGFR mRNA and protein in bladder cancer and normal bladder tissue. A:** LRIG1 and EGFR mRNA expression in bladder cancer with different tumor (T) stages and normal bladder tissue. ^*^P < 0.05 vs normal tissue. ^#^P < 0.05 vs T1 stage. **B**: Immunohistochemical analysis of LRIG1 and EGFR expression in bladder cancer with different tumor (T) stages and normal bladder tissue.

In order to examine the mRNA expression of LRIG1 and EGFR in bladder cancer, 45 tumor RNA samples and corresponding 5 normal tissues RNA samples were analyzed by quantitative real-time RT-PCR. Compared with corresponding nonneoplastic tissue, the expression of LRIG1 appeared downregulated in all of the tumor (Figure 1A). Meanwhile, the expression of EGFR was elevated in all of the tumor compared to the mean in the respective non-neoplastic tissue (Figure 1A). Next, expression of LRIG1 and EGFR protein were determined by IHC. IHC staining also demonstrated downregulation of LRIG1 protein in bladder cancer tissue (Figure 1B). Then we compared the expression of LRIG1 and EGFR in different stage. We found that the LRIG1 expression in T2-T3 stage were significantly lower than that in T1 stage. This phenomenon could indicate that the expression of LRIG1 were lower in aggressive bladder cancer.

### EGFR was negatively regulated by LRIG1 on bladder cancer cells

**Figure 2:**
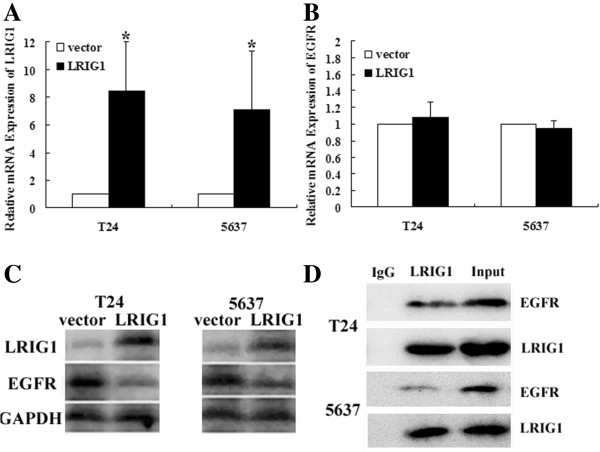
**The effect of LRIG1 transfection on expression of EGFR. A:** The mRNA expression of LRIG1 was examined by real time-PCR after transfection. **B:** The mRNA expression of EGFR was examined by real time-PCR after transfection. **C:** The protein expression of LRIG1 and EGFR was examined by western blot after transfection. Upregulation of LRIG1 significantly decreased endogenous EGFR protein. **D:** Lysates were immunoprecipitated with rabbit anti-LRIG1 or control IgG and blotted with antibodies to EGFR or LRIG1 (*P < 0.05).

The plasmid p3XFLAG-CMV9-LRIG1 was transfected into T24 and 5637 cells to analyze whether LRIG1 might be a functional regulator of EGFR. Effects of LRIG1 gene transfection on EGFR expression in transcription and translation level were examined by quantitative real-time RT-PCR and Western blotting method with their respective primer and antibodies. We observed that LRIG1 gene transfection did not have an impact on the endogenous EGFR mRNA level, but upregulation of LRIG1 was followed by a substantial decrease in the protein level of EGFR (Figure 2B,C). It can be inferred that upregulation of LRIG1 may directly impact EGFR protein, but not via transcription regulation.

Because upregulation of LRIG1 only impact the protein level of EGFR, subsequently a co-immunoprecipitation method was used to determine whether there was a physical interaction between LRIG1 and EGFR molecules. We observed that EGFR could be specifically co-immunoprecipitated with LRIG1, but not with control IgG, indicating that two proteins are specifically associated in complex with each other (Figure 2D).

### LRIG1 inhibited cell growth in bladder cancer cells

It was reported previously that inhibition of EGFR signaling could induce apoptosis and inhibit growth of tumor cells [17,18]. We concluded that upregulation of LRIG1 could induce the same impact.

**Figure 3:**
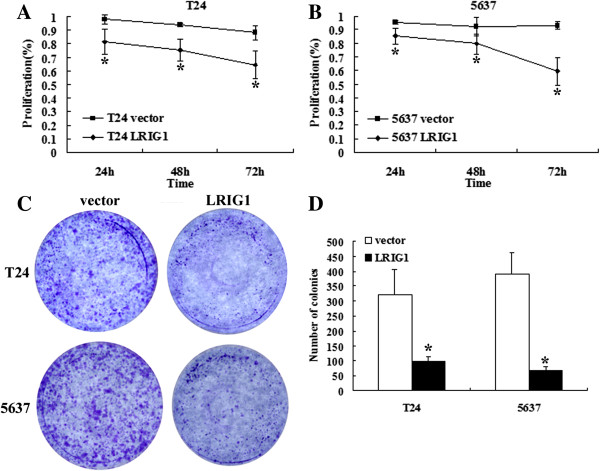
**Effect of LRIG1 gene transfection on growth of human bladder cancer cells. A:** LRIG1 gene transfection could inhibit T24 proliferation by cck-8 assay(*P < 0.05). **B:**LRIG1 gene transfection could inhibit 5637 proliferation by cck-8 assay (*P < 0.05). **C:** LRIG1 gene transfection could inhibit cell viability by quantitative clonal forming assay. **D:** Data showed transfection of LRIG1 cDNA could significantly inhibit the cell viability as compared with vector cells (*P < 0.05). All experiments were repeated at least three times.

CCK-8 assay revealed that the proliferation of T24 and 5637 cells transfected with LRIG1 cDNA was remarkably decreased, compared to the corresponding vector control (P < 0.05) (Figure 3A,B). These results were further supported by a quantitative clonal forming assay. Transfection of T24 and 5637 cells with LRIG1 cDNA could inhibit cell viability, which would lead to a significant decrease of the number of colonies compared with vector and control cells (P < 0.05) (Figure 3C,D).

### LRIG1 induced apoptosis and reversed invasion in bladder cancer cells

**Figure 4:**
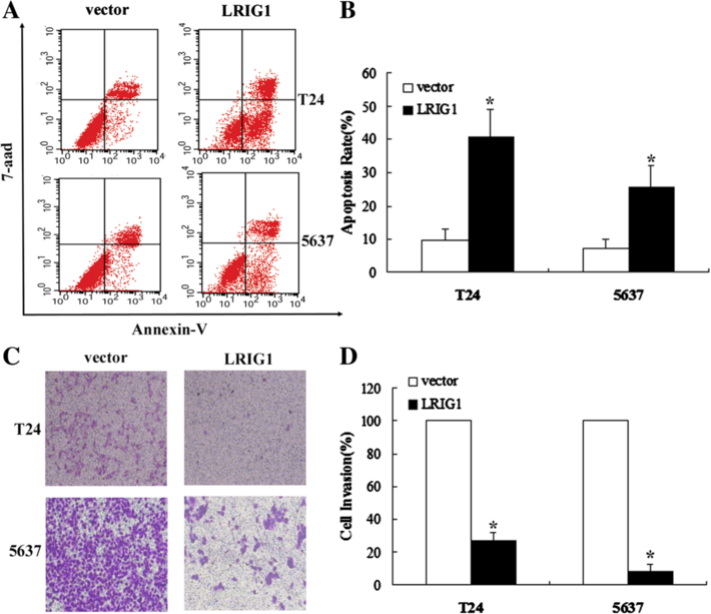
**LRIG1 gene transfection induced apoptosis and inhibit invasion in bladder cancer cells. A**: LRIG1 gene transfection induced apoptosis in human T24 and 5637 cell lines by flow cytometry analysis. **B**: The percentages are displyed showing the annexin V positive/7-aad negative fraction. Columns are expressed as mean ± SD of three independent experiments. *P < 0.01 for LRIG1 cDNA versus vector. **C:** Effect of LRIG1 gene transfection 24 h on the cell invasion of human bladder cancer cells. **D:** Data showed transfection of LRIG1 cDNA could significantly inhibit the cell invasion as compared with vector cells (*P < 0.05). All experiments were repeated at least three times.

The apoptotic effect of LIRG1 on bladder cancer cell lines was detected through Annexin V-PE/7-aad double staining assay (Figure 4A,B). Stained cells were immediately analyzed by flow cytometry. Results demonstrated that LRIG1 overexpression has an effect on increasing apoptosis. With Annexin V-PE staining, early apoptosis was clearly detectable in the two bladder cancer cells treated with transfection of LRIG1. Compared to the corresponding vector control, the cell apoptotic rates of LRIG1 were significantly increased in the two cells (P < 0.05).

We next detected whether LRIG1 regulated cell invasion and motility by using the Matrigel in vitro invasion assay. As shown in Figure 4C,D, LRIG1 cDNA exerted a profound effect on cell invasion in the two bladder cancer cells. Compared with the vector and control cells, the T24 and 5637 cells transfected with LRIG1 cDNA, showed a considerably lower invasion potential. These observations indicated that the enhanced expression of LRIG1 was associated with reversed invasive ability.

### Effect of LRIG1 gene transfection on EGFR signaling

**Figure 5:**
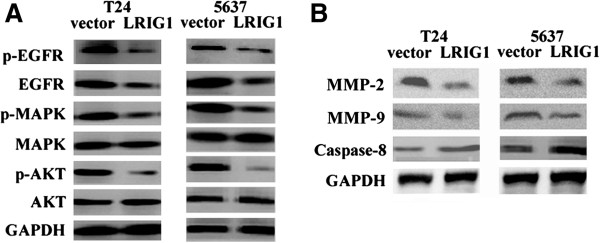
Effect of LRIG1 gene transfection on protein expression of several key regulators involved in the EGFR signaling pathway (A), caspase-8, MMP-2 and MMP-9 (B) of T24 and 5637 cells.

To further demonstrate overexpression of LRIG1 inducing the observed growth inhibition and apoptosis that might correlate with downstream EGFR signaling, we examined the effect of LRIG1 gene transfection on the expression of several key regulators involved in the EGFR signaling pathway. As shown in Figure 5A, western blot analysis detected that upregulation of LRIG1 resulted in a significant reduction in phosphorylation of EGFR (p-EGFR) and EGFR in T24 and 5637 cells. The level of activated mitogen-activated protein kinase (p-MAPK), a downstream regulator of EGFR signaling, showed remarkable decrease in the face of upregulation of LRIG1. Downregulation of p-AKT expression was also observed with LRIG1 cDNA transfection, compared with the vector control.

Caspases represent central regulators of apoptosis. we examined the levels of the active form of caspase-8 to detect the apoptotic response. As shown in Figure 5B, compared with the vector control, the expression of active (cleaved) caspase-8 in the two bladder cancer cells was significantly increased treated with LRIG1 gene. We next measured the level of MMP-2 and MMP-9 in this two bladder cancer cells. Treatment with LRIG1 cDNA caused a significant decrease in MMP-2 and MMP-9 Which involved in reversed invasion induced by LRIG1.

### Effect of EGFR knockdown on LRIG1-induced cell proliferation and signal pathway regulation

**Figure 6:**
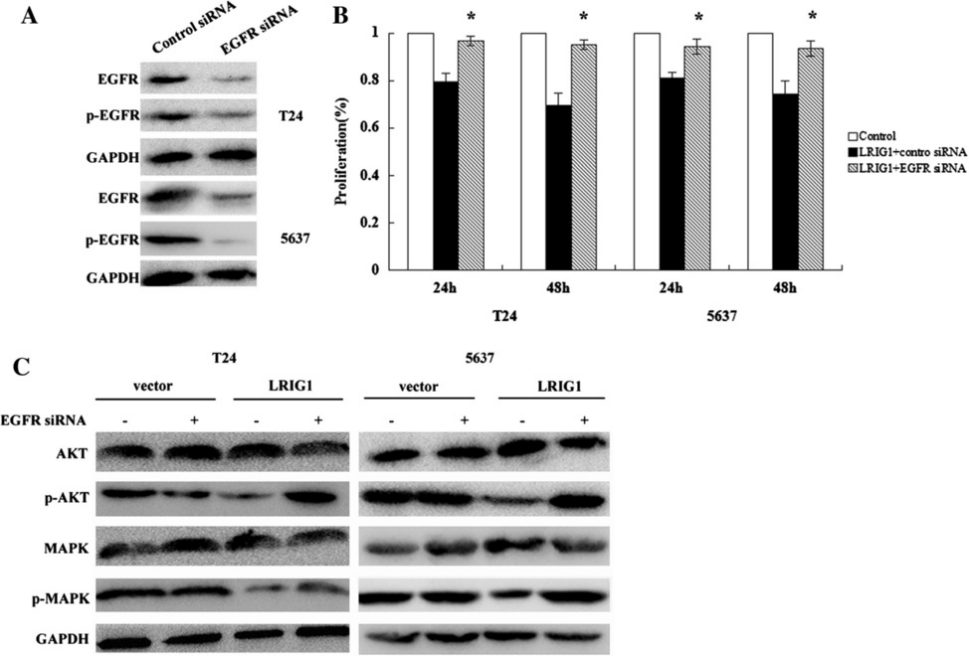
**Effect of EGFR knockdown on LRIG1-induced cell proliferation and signal pathway regulation. A:** Genetic suppression of EGFR by EGFR-siRNA transfection. **B:** Proliferation of cells treated with LRIG1 cDNA after transfection with EGFR siRNA or control siRNA. ^*^P < 0.05 vs cells transfected with control siRNA. **C:** Effects of silencing EGFR on the LRIG1-induced regulation of the expression of AKT, MAPK, and their phosphorylated forms.

To determine whether EGFR expression is critical for the effect of LRIG1 on bladder cancer cells in vitro, we next used specific genetic inhibition of EGFR to assess the consequences of its inhibition on LRIG1 mediated cell proliferation and signal pathway regulation. First, we confirmed that the EGFR siRNA effectively reduced the EGFR protein level in T24 and 5637 cells (Figure 6A). Then we found EGFR knockdown significantly decreased the effect of LRIG1 cDNA on cell proliferation compared with control-siRNA-transfected cells (Figure 6B). And EGFR siRNA significantly weakened the effect of LRIG1 cDNA on the EGFR signaling pathway regulation in both cell lines compared with cells transfected with control siRNA (Figure 6C).

## Discussion

Kekkon proteins negatively regulate the epidermal growth factor receptor (EGFR) during oogenesis in Drosophila. Their structural relative in mammals, LRIG1, is a transmembrane protein, could restrict growth factor signaling by enhancing receptor ubiquitylation and degradation [13]. The feasibility and efficacy of the inhibitory effects of LRIG1 on tumor through inhibiting EGFR signaling activity have been studied in renal cancer, glioma, squamous cell carcinoma of skin, colorectal cancer and prostate cancer [19-23]. In this study, we attempted to evaluate the inhibitory effects of LRIG1 on aggressive bladder cancer cells.

EGFR is a well-studied, versatile signal transducer that is overexpressed in many types of tumour cells, including lung, colon and prostatic carcinoma, and up-regulation of EGFR is associated with poor clinical prognosis [24,25]. EGFR is a 170 kDa tyrosine kinase receptor consisting of an extracellular ligand-binding domain, a transmembrane lipophilic domain, and an intracellular tyrosine kinase domain and the C-terminus region with multiple tyrosine residues [26]. EGFR mediates signals that stimulate proliferation, migration, and metastasis in many tumour types [25,27], and its signal transduction is regulated by stimulatory and inhibitory inputs. LRIG1, whose extracellular region was organized with leucine-rich repeats (LRRs) and immunoglobulin-like domains homologous to mammalian decorin and the Drosophila Kekkon-1 gene, antagonizes the activity of epidermal growth factor receptor family receptor tyrosine kinases and acts within a framework of a negative feedback loop [28]. In our study, we found that the expression of LRIG1 was decreased, whereas the expression of EGFR was increased in bladder cancer tumor versus non-neoplastic tissue. This finding suggest that the downregulation of the LRIG1 gene may be involved in the development and progression of the bladder cancer.

In order to detect the relationship between LRIG1 and EGFR on bladder cancer cells, we examined the expression level of EGFR on T24 and 5637 cells after transfection of LRIG1 cDNA. We observed that up-regulation of LRIG1 did not have an impact on the endogenous EGFR mRNA level, but it was followed by a substantial decrease in the protein level of EGFR. It was reported that upregulation of LRIG1 transcript and protein upon EGF stimulation, and physical association of the encoded protein with the four EGFR orthologs of mammals [13]. As we known, LIRG1 could enhance the ligand stimulated ubiquitination of ErbB receptors in a c-Cbl dependent manner [14]. Cbl-mediated receptor ubiquitylation marks the onset of attenuation. The previous study indicates that overexpression of Cbl in cells promotes EGF-stimulated receptor ubiquitylation and degradation [29].

In the following study, we concluded that upregulation of LRIG1 could induce cell apoptosis and suppress cell growth, and furthermore reverse cell invasion in T24 and 5637 cells. All of this changes of biological behavior suggest that LRIG1 is a tumor suppressor gene on aggressive bladder cancer cells. However, the change of biological behavior is not exclusively attributed to the restriction of one molecule, as the signal transduction is a complicated matter in cells [21,30]. In our study, we examined the effect of LRIG1 gene transfection on the expression of several key regulators involved in the EGFR signaling pathway, including MAPK and AKT. We found that p-MAPK and p-AKT in T24 and 5637 cells were significantly reduced following LRIG1 cDNA transfection which also inhibited phosphorylation of EGFR. Because of the above results we can conclude that LRIG1 indeed affects the biology behaviors of baldder cancer cells in vitro by inhibiting phosphorylation of EGFR and the downstream signaling pathway. And we found that EGFR expression is critical for the effect of LRIG1 on bladder cancer cells in vitro. Taken together, these results could offer a novel therapeutic strategy for suppression of bladder cancer by restoration of LRIG1.

## Competing interests

The authors declare that they have no competing interests.

## Authors’ contributions

LC, RS, TY performed the experiments. FL, GL, YG analyzed the data. BL, WY Contributed reagents/materials/analysis tools. LC, HX Wrote the manuscript. HX, QZ, WY conceived and designed the experiments. All authors read and approved the final manuscript.
